# ARHGAP21 Is Involved in the Carcinogenic Mechanism of Cholangiocarcinoma: A Study Based on Bioinformatic Analyses and Experimental Validation

**DOI:** 10.3390/medicina59010139

**Published:** 2023-01-10

**Authors:** Zhihuai Wang, Siyuan Wu, Gaochao Wang, Zhen Yang, Yinjie Zhang, Chunfu Zhu, Xihu Qin

**Affiliations:** 1 Department of General Surgery, The Affiliated Changzhou No. 2 People’s Hospital of Nanjing Medical University, Changzhou 213000, China; 2 Graduate School, Nanjing Medical University, Nanjing 211166, China

**Keywords:** ARHGAP21, proliferation, migration, PI3K/Akt signaling pathway, immune infiltration, cholangiocarcinoma

## Abstract

*Background and Objectives:* Rho GTPase-activating protein (RhoGAP) is a negative regulatory element of Rho GTPases and participates in tumorigenesis. Rho GTPase-activating protein 21 (ARHGAP21) is one of the RhoGAPs and its role in cholangiocarcinoma (CCA) has never been disclosed in any publications. *Materials and Methods:* The bioinformatics public datasets were utilized to investigate the expression patterns and mutations of ARHGAP21 as well as its prognostic significance in CCA. The biological functions of ARHGAP21 in CCA cells (RBE and Hccc9810 cell) were evaluated by scratch assay, cell counting kit-8 assay (CCK8) assay, and transwell migration assay. In addition, the underlying mechanism of ARHGAP21 involved in CCA was investigated by the Gene Ontology (GO) and Kyoto Encyclopedia of Genes and Genomes (KEGG) enrichment analysis, and the most significant signaling pathway was identified through gene set enrichment analysis (GSEA) and the Western blot method. The ssGSEA algorithm was further used to explore the immune-related mechanism of ARHGAP21 in CCA. *Results:* The ARHGAP21 expression in CCA tissue was higher than it was in normal tissue, and missense mutation was the main alteration of ARHGAP21 in CCA. Moreover, the expression of ARHGAP21 had obvious differences in patients with different clinical characteristics and it had great prognostic significance. Based on cell experiments, we further observed that the proliferation ability and migration ability of the ARHGAP21-knockdown group was reduced in CCA cells. Several pathological signaling pathways correlated with proliferation and migration were determined by GO and KEGG analysis. Furthermore, the PI3K/Akt signaling pathway was the most significant one. GSEA analysis further verified that ARHGAP21 was highly enriched in PI3K/Akt signaling pathway, and the results of Western blot suggested that the phosphorylated PI3K and Akt were decreased in the ARHGAP21-knockdown group. The drug susceptibility of the PI3K/Akt signaling pathway targeted drugs were positively correlated with ARHGAP21 expression. Moreover, we also discovered that ARHGAP21 was correlated with neutrophil, pDC, and mast cell infiltration as well as immune-related genes in CCA. *Conclusions:* ARHGAP21 could promote the proliferation and migration of CCA cells by activating the PI3K/Akt signaling pathway, and ARHGAP21 may participate in the immune modulating function of the tumor microenvironment.

## 1. Introduction

Cholangiocarcinoma (CCA) is a diverse epithelial cell malignancy, and the overall morbidity of CCA has gradually augmented at home and abroad over the past decades [1]. CCA takes up nearly 3% of digestive malignancies and 15% of all primary liver cancers nowadays. Known as the second most common liver malignancy after hepatocellular carcinoma (HCC) [2], CCA has attracted expanded attention due to its low diagnostic rate, dismal prognosis, and high fatality rate [3,4,5,6]. Although great progress has been achieved in mechanisms and management in recent years [7,8], which included aspects of epidemiology, molecular pathogenesis, diagnosis, therapy, cell survival signaling pathways, and tumor microenvironment [9], the pathogenesis of most CCA is still unclear and the therapy of CCA is deficient and unsatisfied. In consequence, exploring effective biomarkers and identifying novel targets is indispensable. 

ARHGAP21 is part of the Rho GTPase-activating protein (RhoGAP) family and is highly expressed in differentiated hematological cells and highly differentiated tissues, such as the brain, heart muscle, and placenta. ARHGAP21 acts crucially in multiple cellular processes [10]. As a protein-coding gene, one of the most important roles for ARHGAP21 is reorganizing the cytoskeleton. The cytoskeleton is mainly composed of microtubules, microfilaments, and intermediate fibers. Previous studies generally regarded the cytoskeleton as a supporting structure, but recently, there was a new understanding of the cytoskeleton, in which researchers found that the cytoskeleton and its reorganization are involved in many cellular biological processes. For instance, ARHGAP21 can modulate the cytoskeleton by interacting with Rho-associated kinase (ROCK) and mDia by activating the actin-associated proteins 2/3 (Arp2/3) [11]. ARHGAP21 is also essential for epithelial-mesenchymal transition (EMT) and modulates the acetylation of α-tubulin in cell–cell adhesion formation and the cellular migration process [12]. It was identified that ARHGAP21 was differentially expressed and played different roles in multiple human cancers. For example, ARHGAP21 was considered a RhoGAP for RhoA, and RhoC contributed to the proliferation and migration of prostate adenocarcinoma cells [13]. Bigarella et al. [14] found that ARHGAP21 modulated FAK activities and impaired the migration of glioblastoma cells. Moreover, the function of ARHGAP21 is not only reflected in the effect on cancer but also acts significantly in the regulation of glucose homeostasis, intracellular Golgi transport, and viral replication. Although many studies have described a variety of important biological functions of ARHGAP21 in cancers, its role in CCA has not been reported.

This study focused on the biological effects of ARHGAP21 on CCA cells and its possible mechanism. Notably, it was the first time that ARHGAP21 was found to have the capacity to regulate the proliferation and migration of CCA cells through in vitro cell experiments, which made up for the blank in the field of biological effects of ARHGAP21 on CCA. In addition, this study further explored the mechanism by which ARHGAP21 plays biological roles in CCA cells, and focused on the correlation between ARHGAP21 and immune infiltration. These provide the clues and bases for the in-depth understanding of the biological behavior of ARHGAP21 on CCA. In conclusion, ARHGAP21 may have the potential to become a prognostic biomarker and an effective therapeutic target for CCA in the time to come.

## 2. Materials and Methods

### 2.1. Bioinformatics Data

The data of CCA in gene expression were acquired via the TCGA database (http://tcga-data.nci.nih.gov/tc.ga, accessed on 1 July 2022) and the GEO database (https://www.ncbi.nlm.nih.gov/geo/, accessed on 1 July 2022). The format of the downloaded RNA sequencing data is HTSeq-FPKM. The single gene difference analysis (paired *t*-test and unpaired *t*-test) was performed, and the mRNA expression level of ARHGAP21 in tumor tissues and para-carcinoma tissue was displayed by scatter plot. The ROC curve was drawn to explore the diagnostic value in CCA patients. The relationship between patients’ prognoses and ARHGAP21 among CCA patients was analyzed by using the survminer (0.4.9) and survival (3.2–10) packages in R (3.6.3) software. The data resource is derived from the RNA sequencing data and clinical information of the TCGA online platform. 

### 2.2. Human Protein Atlas

In order to further analyze the differential protein expression of ARHGAP21, we applied the human protein atlas website to explore the staining intensity of ARHGAP21, of which the website is https://www.proteinatlas.org/, accessed on 1 July 2022. The online database included plenty of antibody-based imaging, which can show the distribution and expression of different proteins in various human organs and tissues [15]. Especially the ‘pathology’ module can reveal the protein expression in different human tumors and the impact on patient survival [16]. 

### 2.3. cBioPortal for Cancer Genomics

The cBioPortal for Cancer Genomics online website was applied to explore the mutation frequency of ARHGAP21 in three kinds of cancer resources. It integrates three CCA datasets including CCA (National Cancer Center of Singapore, Nat Genet 2013), CCA (National University of Singapore, Nat Genet 2012), and CCA (TCGA, Firehose Legacy) to conduct an analysis of the mutations on ARHGAP21. Meanwhile, it also serves to analyze the relations between the mutations and clinical parameters. The online website (https://www.cbioportal.org/, accessed on 1 July 2022) is derived from more than two hundred studies and the whole of TCGA data and some published studies; it integrates differential datasets to reflect the panorama of genomics at the gene level, and it specifically analyzes the blueprint of mutations, targeted gene expression, and copy numbers in different tumors [17]. 

### 2.4. GEPIA Online Website

The GEPIA online database serves as an online vehicle in terms of conducting an analysis of gene expression patterns and prognostic significance in human cancers (http://gepia.cancer-pku.cn/detail.php, accessed on 1 July 2022) [18]. This study used it to analyze the expression patterns of AAAS, ACVR1B, ACVRL1, ADAMTS12, and ADCY2 in CCA tissues and normal tissues.

### 2.5. LinkedOmics Database 

The LinkedOmics database is an online platform that can analyze the multi-omics genomic data from 32 human cancer types which include 11,158 patients in the TCGA dataset [19]. The website assisted in analyzing the co-expressed genes of ARHGAP21. The upper 50 genes which were positively or negatively co-expressed with ARHGAP21 were downloaded from the website. The correlation was analyzed by Pearson’s correlation analysis.

### 2.6. GO Enrichment Analysis and KEGG Enrichment Analysis

The DAVID website was employed to deal with the GO enrichment analysis and the KEGG enrichment analysis for genes, to which the ARHGAP21 was evidently negatively or positively related. The DAVID website reveals the enriched functions and the enriched signaling pathways of a group of genes.

### 2.7. Cell Lines and Cell Culture

Human CCA cell line HUCCT1 was bought from Shanghai Fuxiang Biotechnology Co.; Ltd. Human CCA cell lines Hccc9810 and RBE were bought from Guangzhou Huatuo Biotechnology Co., Ltd. All cells were cultivated in Roswell Park Memorial Institute-1640 medium (Gibco, Grand Island, NY, USA) containing 10% fetal bovine serum (Gibco, Lofer, Austria), 100 U/mL penicillin, and 100 U/mL streptomycin, and then were incubated in a humidified atmosphere with 5% CO_2_ in air at 37 °C.

### 2.8. RNA Extraction and qRT-PCR Analysis

Cells collected and the total RNA samples extracted were from CCA cell lines using a Total RNA TriPure Isolation Reagent Kit (BioTeke, Beijing, China) according to the manufacturer’s protocol. RNA was synthesized to first-strand cDNA using the HiscriptII Q RT SuperMix for qPCR with gDNA wiper (R223-01; Vazyme Biotech, Nanjing, China). The reactions were performed for 15 min at 42 °C in a TRIO Thermoblock (Biometra, Goettingen, Germany). Quantitative real-time PCR (qRT-PCR) was performed by an AceQ qPCR SYBR Green Master Mix Kit (Q131-02; Vazyme Biotech, Nanjing, China) in an ABI 7500 RT-PCR system (Applied Biosystems, Foster City, CA, USA) using the following parameters: 1 cycle of pre-denaturation at 95 °C for 10 min, followed by 40 cycles at 95 °C for 10 s, 60 °C for 30 s, and 70 °C for 10 s. At this point, mRNA expression was normalized to GAPDH and the relative fold changes were calculated using the 2^−ΔΔCt^ method. Each sample was run independently in triplicate. The primers for the ARHGAP21 and GAPDH sequences are listed in Table 1.

### 2.9. Cell Transfection

CCA cells were seeded in six-well plates and transfected by siRNA and negative control (NC) at a final concentration of 50 nM using a transfection reagent (RiboBio). SiRNA and NC were purchased from RiboBio (Guangzhou, China). All transfections were performed pursuant to the manufacturer’s instructions. At 48 h after transfection, the transfection solution was completely substituted by the culture medium. Cells for extracting RNA were harvested after being treated for 48 h, following the manufacturer’s recommendations. The transfection efficiencies of siRNA were determined by qRT-PCR.

### 2.10. Cell Counting Kit-8 (CCK-8) Assay

The measurement of the liveness of the cell was done by CCK-8 reagent (Beyotime Technology, Beijing, China) following the manufacturer’s scheme: seed at a density of 2000 cells per well in a 96-well plate. The measurement of the cell viability was conducted once per 24 h till day 4. The experiment was performed in triplicate.

### 2.11. Cell Scratch Assay

First and foremost, seed CCA cells onto 6-well plates until they reached proper confluency. Secondly, scratch each well with a sterile 100-UL pipette tip to create three to five artificial homogeneous wounds and cultivate the remaining cells in a serum-free medium. Last but not least, capture the migration of the cell with an inverted microscope respectively at 0 h and 48 h.

### 2.12. Transwell Migration Assay

In this process, we seeded 3 × 10^4^ BC cells in a serum-free medium in the upper chamber. Meanwhile, the lower chamber was added with a 10% FBS medium. The temperature of the incubation of the cells was 37 °C and the duration of the incubation was 24 h. A microscope (Nikon Eclipse 80i) was used to count the migrating cells after they were fixed and stained.

### 2.13. Western Blot Assay

Cells were collected and lysed in RIPA lysis buffer and were then added with 1:50 protease and phosphatase inhibitors on ice for 2 min. Use a BCA reagent (Beyotime, Beijing, China) to determine the protein concentration after centrifuging the specimen at 12,000× *g* for 10 min at 4 °C. Then submit the same quantity of protein to 6% or 8% sodium dodecyl sulfate-polyacrylamide gel electrophoresis. After that, transfer it to polyvinylidene fluoride membranes (Millipore Corp.; Bedford, MA, USA). Block the membranes by QuickBlockTM Blocking Buffer for Western blot at room temperature for 1 h and then incubate them with the primary antibodies against p-Akt (1:2000 dilution), ARHGAP21 (1:1000 dilution), Akt (1:1000 dilution), p-PI3K (1:2000 dilution), PI3K (1:1000 dilution), and β-Tubulin (1:1000 dilution) at 4 °C overnight. In our experiment, the protein levels were normalized to β-Tubulin. The blots were then labeled with the anti-rabbit-mouse IgG secondary antibody (1:10,000 dilution) for 0.5 h at 37 °C. Detect the protein bands with an enhanced chemiluminescence reagent (Thermo Fisher Scientific, Waltham, MA, USA). 

### 2.14. TIMER

The TIMER online website was adopted to analyze the correlations between infiltrating immune cells and ARHGAP21 expression in the tumor microenvironment, whose website is http://timer.cistrome.org/, accessed on 1 July 2022. The TIMER online database focuses on immune-related research of human malignant tumors; it also analyzes and exports the immunologic and genomics information of about thirty-two types of tumors [20]. 

## 3. Statistical Analysis

The data were all obtained from no less than three separate trials and were proceeded to statistical analysis with GraphPad Prism 8.2.1. In three parallelly conducted trials, the analysis of the differentiation in the statistics was conducted by either *t*-test or one-way analysis of variance (ANOVA). All values were expressed as mean ± standard deviation (SD). Under this circumstance, *p* < 0.05 was deemed to make sense from the perspective of statistics. In order to further validate the relationship between ARHGAP21 and the PI3K/Akt signaling pathway, we downloaded the drug response data of Genomics of Drug Sensitivity in Cancer (GDSC) to explore the association between ARHGAP21 and PI3K/Akt signaling pathway inhibitors. The association between ARHGAP21 and immune cells was analyzed through the ssGSEA algorithm of the GSVA package (1.34.0) in R software (3.6.3). The immune cells incorporated cytotoxic cells, eosinophils, DC, iDC (immature DC), aDC (activated DC), CD8 T cells, B cells, NK CD56bright cells, neutrophils, NK cells, NK CD56dim cells, mast cells, macrophages, T cells, pDC (Plasmacytoid DC), T helper cells, Tem (T effector memory), Tfh (T follicular helper), Tcm (T central memory), Tgd (T gamma delta), Th1 cells, Th2 cells, Th17 cells, and Treg25. The correlation in respect of the immune-related genes’ expression and ARHGAP21 expression was analyzed and visualized by employing R software (3.6.3) and the ggplot2 (3.3.3) package.

## 4. Results

### 4.1. The Elevated Expression of ARHGAP21 in CCA

**Figure 1:**
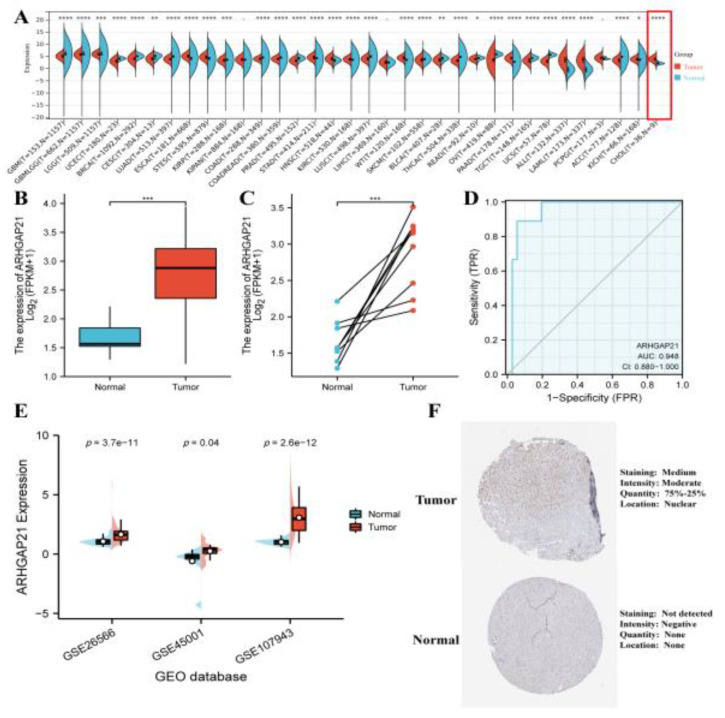
The expression patterns of ARHGAP21 in CCA. (**A**) The ARHGAP21 expression in various cancer. (**B**) The differential mRNA expression of ARHGAP21 in normal tissues and CCA tissues was detected by utilizing unpaired *t*-test analysis in TCGA database. (**C**) The differential mRNA expression of ARHGAP21 was detected in normal tissues and CCA tissues by utilizing paired t-test analysis in TCGA database. (**D**) The ROC curves indicated the diagnostic efficiency of ARHGAP21 in CCA. (**E**). The differential mRNA expression of ARHGAP21 was detected in normal tissues and CCA tissues in three GEO datasets. (**F**). The protein expression of ARHGAP21 between CCA tissue and normal tissue. * *p* < 0.05; ** *p* < 0.01; *** *p* < 0.001; **** *p* < 0.0001.

The expression of ARHGAP21 in various cancers was analyzed and showed that the ARHGAP21 mRNA of tumors was upregulated (*p* < 0.0001, Figure 1A). Especially the ARHGAP21 expression in CCA tissue was elevated compared with normal tissue. Moreover, the data indicated that the level of the mRNA expression of ARHGAP21 in CCA tissue was to a great extent higher than that in normal neighboring tissues (*p* < 0.001, Figure 1B), and the difference was statistically significant. The paired *t*-test showed that ARHGAP21 is highly expressed in CCA (*p* < 0.001; Figure 1C). The ROC curve showed that the diagnostic value of ARHGAP21 in CCA patients was significant (ROC = 0.948, 95CI = 0.880–1.000, Figure 1D). Three GEO datasets were applied to validate the over-expression of ARHGAP21 in tumor tissue (GSE26566: *p =* 3.7 × 10^−11^; GSE45001: *p =* 0.04; GSE107943: *p* = 2.6 × 10^−12^; Figure 1E). The protein expression of ARHGAP21 was statistically elevated in CCA tissue according to the results of the HPA database (Figure 1F).

### 4.2. The Significant Correlation between ARHGAP21 and Clinical Characteristics in CCA Patients

**Figure 2:**
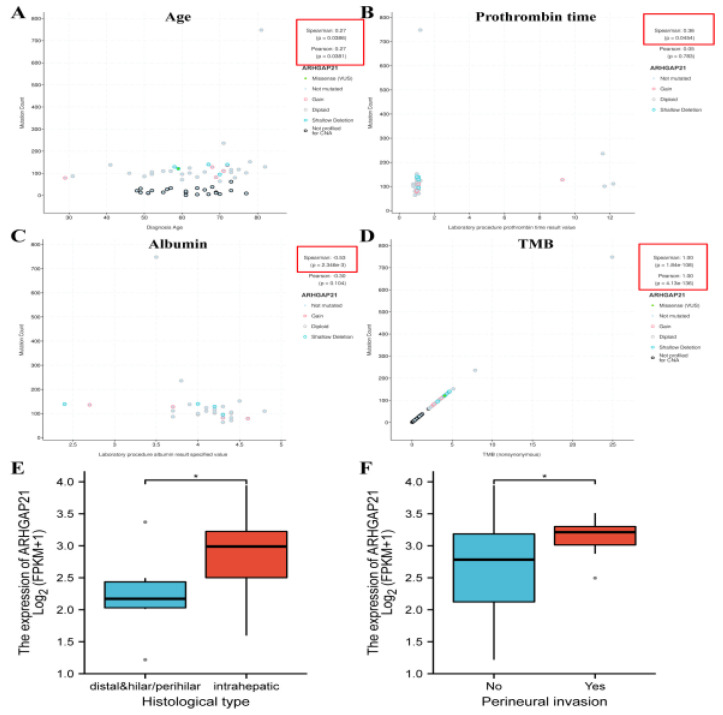
The association of ARHGAP21 and clinicopathological features in CCA patients. (**A**) ARHGAP21 and age. (**B**) ARHGAP21 and prothrombin time. (**C**) ARHGAP21 and albumin (**D**). ARHGAP21 and TMB. (**E**) ARHGAP21 and histological type. (**F**) ARHGAP21 and perineural invasion. * *p* < 0.05.

Patient age is positively correlated with the mutation count of ARHGAP21 (Spearman R = 0.27, *p* = 0.0386; Pearson R = 0.27, *p* = 0.0381; Figure 2A). There is a positive correlation between prothrombin time and the mutation count of ARHGAP21 (Spearman R = 0.36, *p* = 0.0454; Figure 2B), whereas the correlation between albumin and the mutation count of ARHGAP21 (Spearman R = −0.53, *p* = 2.346 × 10^−3^; Figure 2C) is negative. ARHGAP21 mutation is significantly correlated with TMB in CCA (Spearman R = 1, *p* = 1.84 × 10^−8^; Pearson R = 1, *p* = 4.13 × 10^−136^; Figure 2D). According to the analysis of downloaded TCGA-CHOL RNA sequencing data, there was relevance between the expression of ARHGAP21 and the histological type of CCA (*p* < 0.05; Figure 2E), and the high expression of ARHGAP21 occurred in the perineural invasion group (*p* < 0.05; Figure 2F).

### 4.3. The Prognostic Significance of ARHGAP21 in CCA Patients with Multiple Clinicopathological Features

The result showed that ARHGAP is associated with OS in CCA patients with clinical features including the T2 stage (*n* = 12, HR = 0.11, 95 CI = 0.01−0.94, *p* = 0.044; Table 2), T1 + T2 stage (N = 31, HR = 0.25, 95 CI = 0.08−0.77, *p* = 0.016; Table 2), N0 + N1 stage (N = 31, HR = 0.25, 95 CI = 0.07−0.90, *p* = 0.034; Table 2), M0 + M1 stage (N = 33, HR = 0.28, 95 CI = 0.09−0.87, *p* = 0.028; Table 2), TNM stage I + stage II (N = 28, HR = 0.23, 95 CI = 0.06−0.87, *p* = 0.03; Table 2), White race (N = 31, HR = 0.30, 95 CI = 0.09−0.94, *p* = 0.039; Table 2), CA19-9 normal (N = 14, HR = 0.10, 95 CI = 0.01−0.90, *p* = 0.04; Table 2), without vascular invasion (N = 29, HR = 0.26, 95 CI = 0.07−0.96, *p* = 0.042; Table 2), and without perineural invasion (N = 26, HR = 0.19, 95 CI = 0.04−0.88, *p* = 0.034; Table 2). ARHGAP is associated with DSS in CCA patients with clinical features including the T1 + T2 stage (N = 31, HR = 0.28, 95 CI = 0.09−0.90, *p* = 0.033; Table 2), N0 + N1 stage (N = 31, HR = 0.27, 95 CI = 0.07−0.99, *p* = 0.049; Table 2), M0 + M1 stage (N = 33, HR = 0.30, 95 CI = 0.10−0.95, *p* = 0.041; Table 2), TNM stage I + stage II (N = 28, HR = 0.26, 95 CI = 0.07−0.96, *p* = 0.043; Table 2), and without perineural invasion (N = 26, HR = 0.21, 95 CI = 0.04−0.98, *p* = 0.047; Table 2). The nomogram presented the great predictive performance of ARHGAP21 for the OS and DSS of CCA patients (Supplementary Figure S1). 

### 4.4. The Mutations of ARHGAP21 in CCA

**Figure 3:**
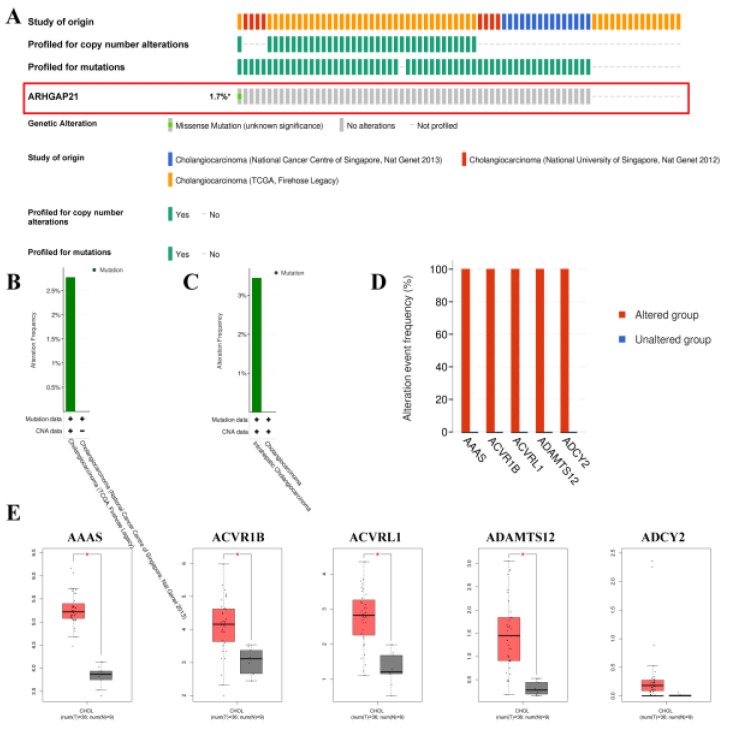
The mutation analysis of ARHGAP21 in CbioPortal online database. (**A**) The alteration map (oncoprint) of ARHGAP21 in CCA database. (**B**) The alteration frequency of ARHGAP21 in different CCA datasets. (**C**) The alteration frequency of ARHGAP21 in differential cancer types. (**D**) The most significant mutated gene between the ARHGAP21 changed group and the unchanged group. (**E**) The differential expression of AAAS, ACVR1B, ACVRL1, ADAMTS12, and ADCY2 between CCA and normal tissue, * *p* < 0.05.

The CbioPortal online website indicated that the total mutation frequency of ARHGAP21 was 1.7% (Figure 3A). The alteration frequency is higher in CCA (TCGA, Firehose Legacy), as shown in Figure 3B. The alteration frequency of intrahepatic cholangiocarcinoma is higher than that in cholangiocarcinoma (Figure 3C). The top five mutated genes (AAAS, ACVR1B, ACVRL1, ADAMTS12, and ADCY2) between the ARHGAP21 changed group and unchanged group were determined and indicated in Figure 3D. The AAAS, ACVR1B, ACVRL1, and ADAMTS12 had a higher level of expression in CCA tissue than that in para-carcinoma tissue (Figure 3E).

### 4.5. Detection of ARHGAP21 Expression of CCA Cell Lines and the Transfection Efficiency of siRNA-ARHGAP21

**Figure 4:**
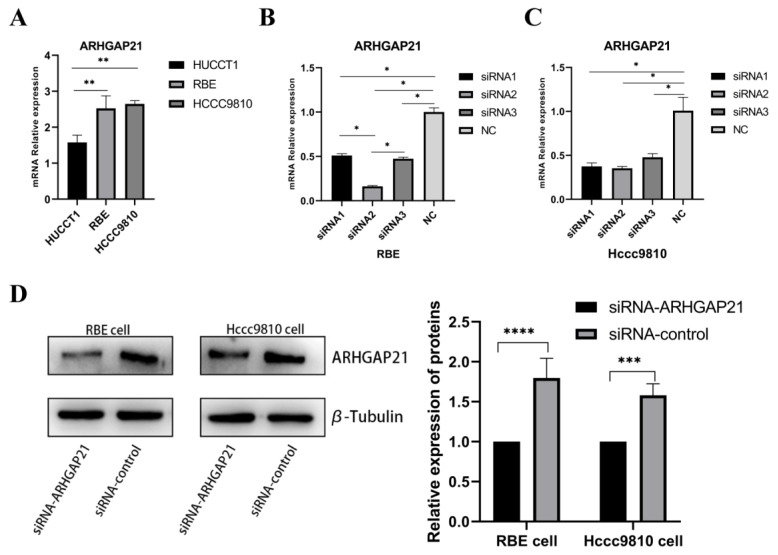
Identification of ARHGAP21 expression in three types of CCA cell lines and exploring the transfection efficiency of siRNA-ARHGAP21 in CCA cells. (**A**) The mRNA expression of ARHGAP21 in three kinds of CCA cells (HUCCT1, RBE, and Hccc9810). (**B**,**C**) The mRNA expression of ARHGAP21 in RBE and Hccc9810 cells after transfecting with three kinds of siRNA-ARHGAP21. (**D**) The protein expression of ARHGAP21 was detected by Western blot assay after transfecting with siRNA2-ARHGAP21. * *p* < 0.05; ** *p* < 0.01; *** *p* < 0.001; **** *p* < 0.0001.

As shown in Figure 4A, the expressions of ARHGAP21 in three strains of CCA cells were detected. The RBE cell line and Hccc9810 cell line were selected to perform the subsequent experiments by advantage of their higher expressions of ARHGAP21 (*p* < 0.01, Figure 4A). Then, three siRNAs were applied in the inhibition of the expressions of ARHGAP21 in Hccc9810 cells and RBE cells. As shown in Figure 4B,C, siRNA2 with a better efficiency in reducing the expression of ARHGAP21 was used to construct ARHGAP21 knockdown cells. Western blot verified the protein expression level of ARHGAP21 after the siRNA2 transfection. As shown in Figure 4D, siRNA2 successfully inhibited the expression of ARHGAP21 levels (*p* < 0.05).

### 4.6. Downregulation of ARHGAP21 Inhibited the Proliferation and the Migration of CCA Cells 

**Figure 5:**
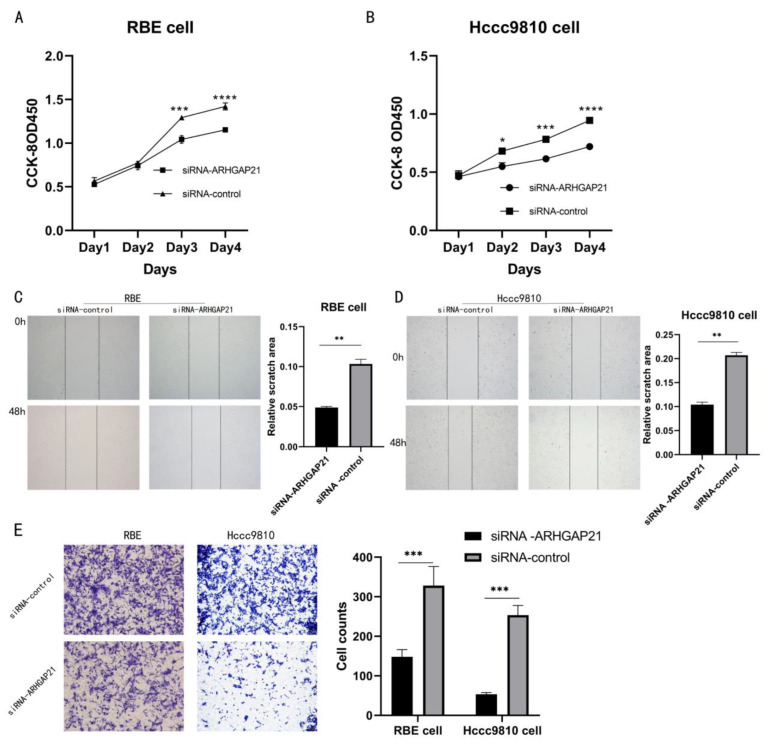
The influence of the proliferation and the migration after transfection with siRNA-ARHGAP21 in CCA cells. (**A**,**B**) The ability of proliferation was detected by CCK-8 assay after transfection with siRNA-ARHGAP21 in RBE cells and Hccc9810 cells. (**C**,**D**) The migration ability of RBE cells and Hccc9810 cells was compared by the percentage of migration area at 0 h and 48 h in the scratch experiment. (**E**) The migration ability of RBE cells and Hccc9810 cells was detected by the transwell migration experiment. Note: the more cells, the stronger the migration ability. * *p* < 0.05; ** *p* < 0.01; *** *p* < 0.001; **** *p* < 0.0001.

After the construction of ARHGAP21 knockdown cells, the cell proliferation assay (CCK-8 assay) result presented that ARHGAP21 depletion inhibited the ability of proliferation in RBE cells (Day 3, *p* < 0.005, Day 4, *p* < 0.001, Figure 5A). In the Hccc9810 cell, the outcome of the CCK-8 assay revealed that the capability of proliferation was also significantly inhibited with ARHGAP21 depletion (Day 2, *p* < 0.05, Day 3, *p* < 0.005, Day 4, *p* < 0.001, Figure 5B). The biological effect of ARHGAP21 was explored by the transwell migration assay and scratch assay. The outcome of the scratch assay showed that the ability of migration in both CCA cells transfected with siRNA-ARHGAP21 was significantly reduced (*p* < 0.01, Figure 5C,D). The outcome of the transwell migration trial indicated that the number of migration cells (148 ± 18.33) in RBE cells in which ARHGAP21 was downregulated was less than the control group (328 ± 48.66; *p* < 0.005, Figure 5E). The ability of migration was likewise inhibited in the Hccc9810 cells where ARHGAP21 was downregulated, and the number of migration cells in the ARHGAP21 downregulated group (53 ± 4.16) was much lower than that in the control group (253 ± 24.44); the differentiation was of statistical significance (*p* < 0.005, Figure 5E).

### 4.7. The Co-Expression Network of ARHGAP21 in CCA

**Figure 6:**
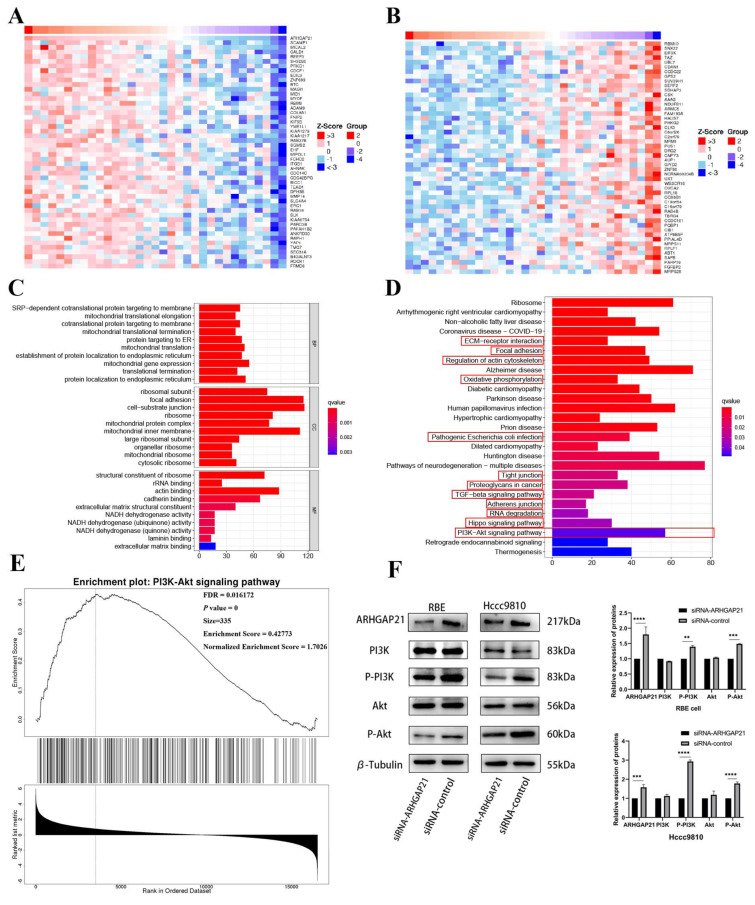
The underlying signaling pathway of ARHGAP21 in CCA. Gene heatmap with positive (**A**) or negative (**B**) correlation with the expression of ARHGAP21. GO enrichment analysis (**C**) and KEGG enrichment analysis (**D**) of the genes related to ARHGAP21. (**E**) The PI3K/Akt signaling pathway was enriched by utilizing GSEA enrichment analysis on Linkedomics online website. (**F**) The protein expression of ARHGAP21, PI3K, P-PI3K, Akt, P-Akt, and β-Tubulin in siRNA-ARHGAP21 group and siRNA-control group of RBE cells and Hccc9810 cells. ** *p* < 0.01; *** *p* < 0.001; **** *p* < 0.0001.

ARHGAP21 can regulate the proliferation and migration of CCA cells per the above results. Therefore, the LinkedOmics website suggested that there were 1486 genes (dark red dots) that highly positively correlated with ARHGAP21, whereas 1020 genes (dark green dots) appeared to be highly negatively correlated with ARHGAP21 (false discovery rate, FDR < 0.05). The top 50 genes that were most strongly correlated with ARHGAP21, whether positively or negatively correlated, were shown in the heat map (Figure 6A,B).

### 4.8. GO and KEGG Enrichment Analysis

The results obtained from the LinkedOmics database indicated that the biological enrichment genes were determined, with which the expression of ARHGAP21 was positively related. The GO analyses were performed, as shown in Figure 6C. There were three biological processes (BP) in which genes positively related to ARHGAP21 expression were involved, including SRP-dependent co-translational protein targeting the membrane, mitochondrial translational elongation, and co-translational protein targeting the membrane. The following three cell components (CC) were what were incorporated in these co-expressed genes: the ribosomal subunit, focal adhesion, and cell-substrate junction. Moreover, three dominating molecular functions (MF) of these co-expressed genes were structural constituents of the ribosome, rRNA binding, and actin binding, respectively. The KEGG pathway analysis indicated that the enriched terms positively connected with the migration function were focal adhesion, ECM receptor interaction, regulation of actin cytoskeleton, oxidative phosphorylation, pathogenic Escherichia coli infection, tight junction, proteoglycans in cancer, TGF-beta signaling pathway, adherens junction, RNA degradation, Hippo signaling pathway, and PI3K/Akt signaling pathway (Figure 6D).

### 4.9. ARHGAP21 Promotes the Proliferation and Migration via PI3K/Akt Pathway in CCA

Based on the above results, it was certain that the most significant pathway which was correlated with migration and proliferation was the PI3K/Akt signaling pathway. The GSEA analysis further confirmed that ARHGAP21 was highly enriched in the PI3K/Akt signaling pathway (*p* = 0, FDR = 0.016172, size = 335, enrichment score = 0.42773; Figure 6E). The Western blot result confirmed that total PI3K protein and total Akt protein expression had no significant difference between the siRNA-ARHGAP21 group and the siRNA-control group in the RBE CCA cell line and Hccc9810 CCA cell line (Figure 6F). However, the phosphorylated PI3K protein and phosphorylated Akt protein decreased significantly in the siRNA-ARHGAP21 group of the RBE CCA cell line and Hccc9810 CCA cell line (*p* < 0.001, Figure 6F).

### 4.10. ARHGAP21 Is Positively Correlated with the PI3K/Akt Signaling Pathway Targeted Drugs

**Figure 7:**
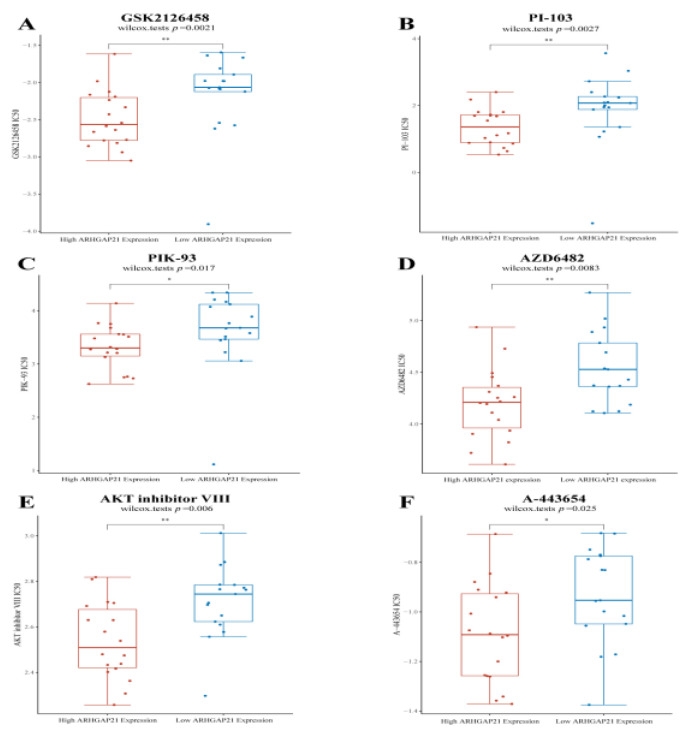
The differential IC50 value of PI3K/AKT pathway targeted drugs between high ARHGAP21 expressed group and low ARHGAP21 expressed group. (**A**) The IC50 value of GSK2126458 (PI3K inhibitor) is different between high ARHGAP21 expressed group and low ARHGAP21 expressed group. (**B**) The IC50 value of PI-103 (PI3K inhibitor) is different between high ARHGAP21 expressed group and low ARHGAP21 expressed group. (**C**) The IC50 value of PIK-93 (PI3K inhibitor) is different between high ARHGAP21 expressed group and low ARHGAP21 expressed group. (**D**) The IC50 value of AZD6482 (PI3K inhibitor) is different between high ARHGAP21 expressed group and low ARHGAP21 expressed group. (**E**) The IC50 value of AKT inhibitor VIII (AKT inhibitor) is different between high ARHGAP21 expressed group and low ARHGAP21 expressed group. (**F**) The IC50 value of A-443654 (AKT inhibitor) is different between high ARHGAP21 expressed group and low ARHGAP21 expressed group, * *p* < 0.05; ** *p* < 0.01.

The results of Genomics of Drug Sensitivity in Cancer (GDSC) further validate the relationship between ARHGAP21 and the PI3K/Akt signaling pathway. The results showed that the IC50 value of PI3K inhibitors were higher in the low ARHGAP21 expression group (GSK2126458, *p =* 0.0021, Figure 7A; PI-103, *p* = 0.0027, Figure 7B; PIK-93, *p* = 0.017, Figure 7C; AZD6482, *p* = 0.0083, Figure 7D). The IC50 value of Akt inhibitors was elevated in the low ARHGAP21 expression group (AKT inhibitor VIII, *p* = 0.006, Figure 7E; A-443654, *p* = 0.025, Figure 7F).

### 4.11. The Strong Relationship of ARHGAP21 and Infiltrating Immune Cells

**Figure 8:**
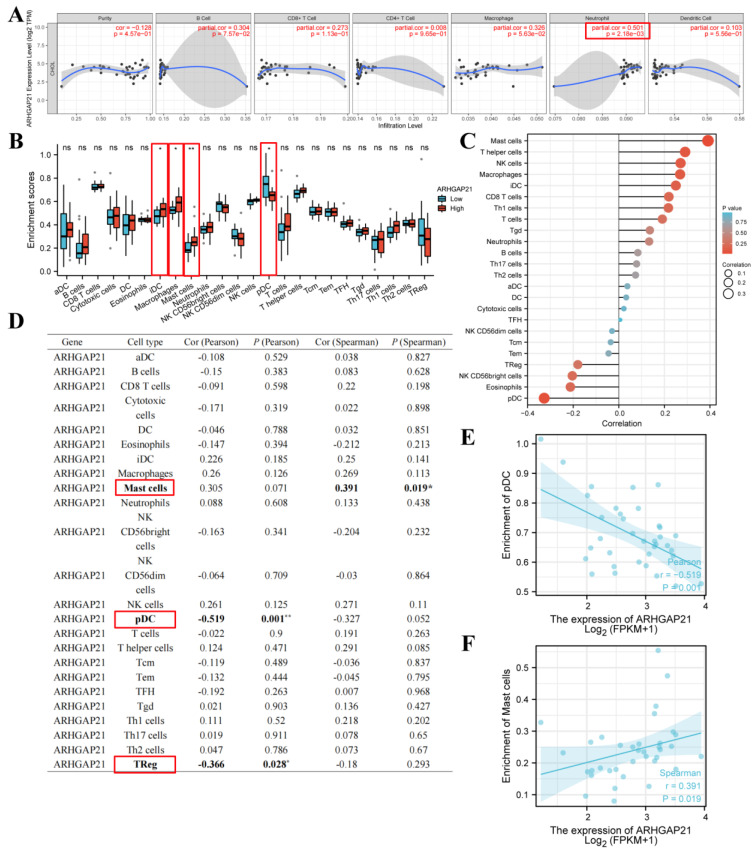
The relationship between ARHGAP21 and immune cells. (**A**) The relationship between ARHGAP21 and immune cells was analyzed by using the TIMER online website. (**B**) The differential infiltration level of immune cells between the high ARHGAP21 group and the low ARHGAP21 group. (**C**,**D**) The Spearman correlation analysis between ARHGAP21 and immune cells was estimated through the ssGSEA algorithm. (**E**) The scatter diagram presented the association of ARHGAP21 and pDC cell. (**F**) The scatter diagram presented the association between ARHGAP21 and mast cells. * *p* < 0.05; ** *p* < 0.01.

Emerging evidence identified that the PI3K/Akt signaling pathway participates in the immune-related mechanism in the carcinogenic process [21]. The above results indicate that ARHGAP21 was correlated with the activation of the PI3K/Akt signaling pathway. Therefore, the relationship between ARHGAP21 and immune cells was analyzed and is presented in Figure 8. ARHGAP21 was significantly correlated with neutrophil infiltration based on TIMER online website (partial cor = 0.501, *p* = 2.18 × 10^−3^; Figure 8A). Through using the ssGSEA algorithm in R software, it was obtained that iDC, macrophages, and mast cells were highly infiltrated in the highly ARHGAP21 expressed cohort (*p* < 0.05; Figure 8B), and pDC was highly infiltrated in the low ARHGAP21 expressed group (*p* < 0.05; Figure 8B). ARHGAP21 had a positive correlation with mast cells (cor = 0.391, *p* = 0.019; Figure 8C,D), and it was negatively correlated with the pDC (cor = −0.519, *p* = 0.001; Figure 8C,D) and Treg (cor = −0.366, *p* = 0.028; Figure 8C,D). Combined with the above results, ARHGAP21 was potentially correlated with pDC (Figure 8E) and mast cells (Figure 8F).

### 4.12. ARHGAP21 Was Correlated with Immune-related Genes

**Figure 9:**
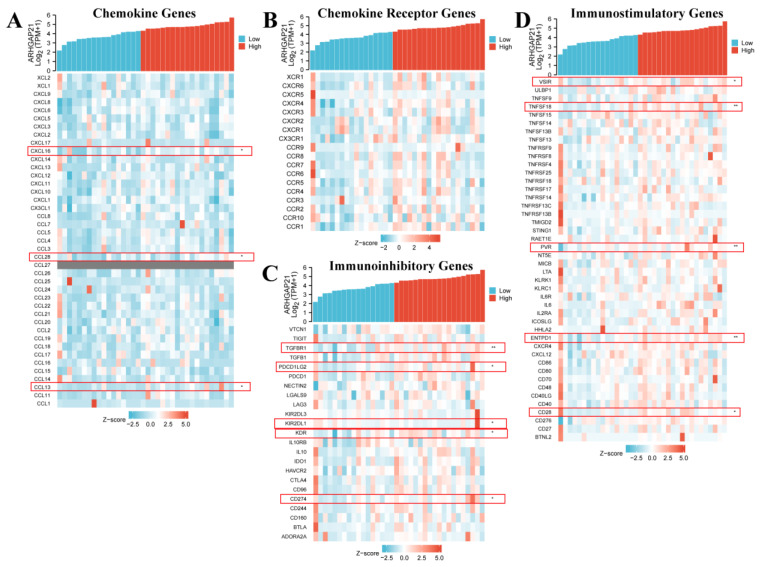
The relationship between ARHGAP21 and immune-related genes. (**A**) The relationship between ARHGAP21 and chemokine genes. (**B**) The relationship between ARHGAP21 and chemokine receptor genes. (**C**) The relationship between ARHGAP21 and immunoinhibitory genes. (**D**) The relationship between ARHGAP21 and immunostimulatory genes. * *p* < 0.05; ** *p* < 0.01.

The outcome of immune-related analysis indicated that ARHGAP21 expression was related to several chemokine genes including CXCL16 (*p* = 0.043, cor = 0.34; Figure 9A and Table 3), CCL28 (*p* = 0.015, cor = 0.403; Figure 9A and Table 3), and CCL13 (*p* = 0.031, cor = 0.359; Figure 9A and Table 3). The chemokine receptor genes have no significant correlation with ARHGAP21 expression (Figure 9B). The immunoinhibitory genes TGFBR1 (*p* = 0.001, cor = 0.53; Figure 9C and Table 3), PDCD1LG2 (*p* = 0.01, cor = 0.425; Figure 9C and Table 3), KIR2DL1 (*p* = 0.02, cor = −0.385; Figure 9C and Table 3), KDR (*p* = 0.023, cor = 0.381; Figure 9C and Table 3), and CD274 (*p* = 0.026, cor = 0.372; Figure 9C and Table 3), and the immunostimulatory genes VSIR (*p* = 0.018, cor = 0.395; Figure 9D and Table 3), TNFSF18 (*p* = 0.002, cor = 0.493; Figure 9D and Table 3), PVR (*p* = 0.003, cor = 0.484; Figure 9D and Table 3), ENTPD1 (*p* = 0.006, cor = 0.45; Figure 9D and Table 3), and CD28 (*p* = 0.02, cor = 0.388; Figure 9D and Table 3) were both associated with ARHGAP21 expression. 

## 5. Discussion

Since its discovery, ARHGAP21 was explored constantly and found it was involved in tumor progression by regulating Rho-GTPase activities. ARHGAP21 is a negative regulator of the Rho-GTPase signaling pathway like other members of the RhoGAP family. It is closely related to actin cytoskeleton dynamics and cell proliferation and differentiation, and has been confirmed to act crucially in the tumorigenesis and development of tumors by regulating Rho-GTPase activity. However, there are few studies about the biological effects of ARHGAP21 on cancer so far, and no article has reported the biological effects of ARHGAP21 on CCA. This is the first exploration of the biological effects of ARHGAP21 on CCA and its mechanism of action; this study fills in the blank in the field of the biological effects of ARHGAP21 on various tumors.

This study proposed that ARHGAP21 may be a potential molecule target of CCA and may take part in the tumorigenesis and development of CCA. The online database analysis verified that ARHGAP21 was upregulated in most human cancers and especially in CCA it is highly expressed. Similar results were also reported in the previous study that ARHGAP21 is overexpressed in prostate adenocarcinoma cells and in head and neck squamous carcinomas [10]. In contrast, Luo et al. [22] obtained the conclusion that ARHGAP21 expression was downregulated in ovarian tumors compared to normal adjacent tissue. Lin et al. [23] reported that ARHGAP21 is also under-expressed in non-small cell lung cancer (NSCLC). Consistent with the above findings, Bass et al. [23] demonstrated that ARHGAP21 was expressed at higher levels in some cancer cell lines, such as HeLaS3 and MOLT4, and at lower levels in others, such as HL-60, A549, and G361 [24]. This suggests that ARHGAP21 may play different roles in different cancers, and it seems to have an important effect on the carcinogenic mechanism of CCA. Interestingly, ARHGAP21 is also overexpressed during myeloid and erythroid differentiation. Studies have shown that ARHGAP21 may be involved in cell differentiation, a procedure that can give rise to cancer when disrupted. It indicated that ARHGAP21 not only contributed to embryonic progression but also directly or indirectly affected cancer progression through some pathways. Furthermore, Bigarella et al. [14] proposed that ARHGAP21 suppressed the cell migration of glioblastoma as the tumor suppressor. Lazarini et al. [13] discovered that ARHGAP21 could regulate the proliferation and the migration of PC3 cells representing advanced prostate cancer, whereas the migration of LNCaP cells representing early prostate cancer was not affected, suggesting that ARHGAP21 may play different roles in different stages of prostate cancer. In summary, ARHGAP21 has a potential effect on different tumorigenesis and tumor progression. In the study, our research found the biological effect of ARHGAP21 on cholangiocarcinoma and identified that it could significantly influence the proliferation and migration ability of CCA cells. This is the first exploration of the biological function of ARHGAP21 in CCA, which was verified by robust cell function experiments in vitro.

The GO enrichment analysis of our research showed three biological processes strongly associated with ARHGAP21: SRP-dependent co-translational protein targeting the membrane, mitochondrial translational elongation, and co-translational protein targeting the membrane. KEGG enrichment analysis of the study showed that ARHGAP21 may have an important relationship with the regulation of the actin cytoskeleton and tight junction. The cytoskeleton is involved in regulating cell shape, motility, transport, and interaction with the environment. Moujaber et al. [25] emphasized that the cytoskeleton plays an important role as it is considered a regulator of cellular signaling pathways. As verified by previous studies, ARHGAP21 regulates cytoskeletal functions by interacting with the crucial cytoskeletal proteins that bind to actin. Meanwhile, our study showed that the inhibition of ARHGAP21 presented a weaker ability for migration in CCA cells. A new hypothesis that ARHGAP21 regulates “Lateral Signaling” explains that there is an interaction between ARHGAP21 and Pk1 and ARHGAP21 also regulates RhoA. Therefore, ARHGAP21 takes control of the volatility of cell shape, which in turn regulates cell migration [26]. Another important interpretation is that the ARHGAP21 depletion reduces the strength of cell–cell adhesion, and as a result, it increases cell migration [12]. The biological function of ARHGAP21 regulating cell migration in CCA was determined by our research, and we attempted to identify the specific signaling pathway of ARHGAP21 influencing the migration of CCA cells in our study. The analyzed results of the Linkedomics website showed that PI3K/Akt is the most significant signaling pathway enriched by ARHGAP21 and its correlated gene. The Western blot confirmed that phosphorylated protein PI3K and Akt expression changed obviously in the ARHGAP21 downregulated group, whereas there was no big difference in the expression of Akt and PI3K. Therefore, we deduced that ARHGAP21 facilitates the migration of CCA cells by regulating the PI3K/Akt signaling pathway. Moreover, the downregulated ARHGAP21 showed a weaker capacity for the proliferation of CCA cells in our study. In previous research, Lazarini et al. [13] verified that ARHGAP21 modulated the proliferation in prostate adenocarcinoma cells, and it might be attributed to the endothelin-1 signaling pathway, of which its most important members are ARHGAP21 partners. This phenomenon explains the possible mechanism by which ARHGAP21 regulates the proliferation of prostate cells. Our finding verified that ARHGAP21 had a positively relevant correlation with the phosphoinositide 3-kinase (PI3K)/Akt signaling pathway and it mediates the proliferation capacity of CCA cells. Therefore, we assume that ARHGAP21 may perform the proliferation function by regulating the PI3K/Akt signaling pathway. The PI3K/Akt pathway acts indispensably in many cellular procedures and its altering frequency in cancer is high, which contributes to the growth and survival of the tumor [27,28]. Akt is one of the most frequently activated kinases in human cancers due to Akt continuously promoting unregulated cell proliferation [29]. In addition, the abnormal Akt signaling is a potential defect in some pathologies [30]. Therefore, our results further determined the effective role of the ARHGAP21 protein in the carcinogenic mechanism of CCA. Meanwhile, our study determined that the drug sensitivity of several PI3K inhibitors and Akt inhibitors were different between the high ARHGAP21 expression group and the low ARHGAP21 expression group. The result suggested that ARHGAP21 seems to activate the PI3K/Akt signaling pathway in the carcinogenic mechanism of CCA and could be a potential therapeutic target. 

Meanwhile, the accumulated research indicates that the PI3K/Akt pathway is involved in cancer immunotherapy. The regulation of the PI3K/Akt signaling pathway can change the cytotoxicity of tumor-infiltrating cells [31]. Gao et al. proposed that the restraint of the PI3K/Akt pathway can inhibit the expression of PD-L1 and enhance the effect against tumors in lung cancer [32]. Zhang et al. verified that tumor-associated macrophage promotes PD-L1 expression through the PI3K/Akt pathway activation in lung cancer [33]. The PI3K/Akt pathway is also reported to regulate the PD-L1 expression in gastric cancer. In this research, we identified that ARHGAP21 influences the migration and proliferation of CCA cells and it induced the cell migration and proliferation via the PI3K/Akt pathway. Therefore, we concentrated on the immune-related mechanism analysis of ARHGAP21 in CCA. Our results showed that ARHGAP expression was negatively correlated with pDC infiltration in the CCA tumor microenvironment. Current studies identified that the infiltration of pDC suggests that immune tolerance exists in the tumor microenvironment and pDC has the potential to be a great prognostic factor for intrahepatic cholangiocarcinoma [34]. Our study found that ARHGAP21 was highly mutated in intrahepatic cholangiocarcinoma from the cBioPortal website and it was negatively correlated with pDC by estimating with the ssGSEA algorithm. Therefore, it indicated that ARHGAP21 may be correlated with the mechanism of immune tolerance and be an effective prognostic biomarker in CCA. González proved that mast cells existed in CCA samples and were involved in the angiogenesis of tumors [35]. Particularly the increased infiltration level of mast cells was detected in advanced CCA patients [36]. Our research indicated that ARHGAP21 expression was positively correlated with mast cell infiltration. Therefore, we supposed that ARHGAP21 may be an essential regulator for angiogenesis and immune infiltration in the CCA tumor microenvironment. In the study, the results also showed that ARHGAP21 is significantly correlated with chemokine genes (CXCL16, CCL28, CL13), immunoinhibitory genes (TGFBR1, PDCD1LG2, KIR2DL1, KDR and CD274), and immunostimulatory genes (VSIR, TNFSF18, PVR, ENTPD1 and CD28). Currently, CXCL16 had to be verified to play an essential role in the carcinogenesis of various cancer and cancer therapy [37]. Tumor hypoxia can activate CCL28 expression to recruit Treg, and induce tumor tolerance [38]. As the main mediator of immune-related processes, TGFBR1 can be a potential predictor for survival in patients with gastric cancer [39]. Levovitz et al. mentioned that the mutations of TGFBR1 can be detected in oropharyngeal cancer [40]. PDCD1LG2 (PD-L2) has been reported to enhance the ability of immune invasion and was expected to be a potential cancer therapeutic target [41]. Current research indicated that CD274 (PD-L1) is expressed on the surface of tumor cells and it can interact with PD-1 which has the potential to reduce the anti-tumor effect [42]. The PD-1/PD-L1 blockade on macrophages can inhibit tumor formation in the mouse model [43]. Increasing evidence suggested that the degradation of PD-L1 combined with the blockade of PD-1/PD-L1 can enhance the effectiveness of cancer therapy [44]. VISTA has been proven to be correlated with the prognosis of pancreatic cancer patients and it may be a great therapeutic target in pancreatic cancer [45]. VSTA is expected to act crucially in tumor-combined immunotherapy [46]. TNFSF18 (GITRL) could mediate NK cell activity and may influence anti-tumor immunity [47]. Chiang et al. mentioned that PVR combined with TIGIT may become a new direction for cancer therapy [48]. Current research indicated that ENTPD1 (CD39) can suppress the anti-tumor activity of T cells and NK cells, which suggests its potential for cancer therapy [49]. CD28 can affect the interaction between PD-1 and PD-L1 and influence T cells’ function in the tumor microenvironment [50]. Recent studies suggested that CD28 could cooperate with CTLA-4 to mediate T-cell activation, and could be an effective target for applying to many immune diseases [51]. Therefore, our findings give strong support for ARHGAP21 being potential immunotherapeutic target in CCA.

In this study, we emphasized the carcinogenesis of ARHGAP21 in CCA tumor development. ARHGAP21 may be an effective immunotherapeutic target and benefit CCA patients in the future. However, some limitations still exist. Due to the limited number of CCA patients, we cannot attain more clinical data to validate the diagnostic and prognostic values of ARHGAP21 based on our clinical samples. The bioinformatics analysis indicated that ARHGAP21 has a significant prognostic value, but it seems to be a protective prognostic factor because the HR value is less than 1. We believe that it is caused by the small CCA sample capacity and we will enroll more clinical samples to obtain a more robust conclusion in the future. Secondly, the specific mechanisms by which ARHGAP21 modulates the PI3K/Akt signaling pathway in CCA cells still need to be further investigated and elucidated. The concrete mechanisms of ARHGAP21 involvement in the CCA microenvironment should be further explored. These will be performed in our future works.

## 6. Conclusions

In summary, the study identified that ARHGAP21 promotes the proliferation and migration of CCA cells via the PI3K/Akt pathway, and it may be the key immune-related regulator in the tumor microenvironment of CCA.

## Figures and Tables

**Table 1:** The primers sequence of ARHGAP21 and GAPDH.

Gene.	Sequence (5′–3′)
ARHGAP21 F	TTGAGCAAACAGCAAACCAG
ARHGAP21 R	GCAACATCTGTTGGTGATGG
GAPDH F	TGAAGGTCGGAGTCAACGGATTTGGT
GAPDH R	CATGTGGGCCATGAGGTCCACCAC

**Table 2:** The prognostic significance of ARHGAP21 in CCA patients who possess various clinical characteristics.

Clinical Characteristic	OS			DSS		
	N	HR	*p*	N	HR	*p*
T stage						
T1	19	0.32 (0.06−1.60)	0.167	19	0.32 (0.06−1.60)	0.167
T2	12	0.11 (0.01−0.94)	**0.044**	12	0.13 (0.01−1.22)	0.074
T3	5	-	**-**	5	-	**-**
T1 + T2	31	0.25 (0.08−0.77)	**0.016**	31	0.28 (0.09−0.90)	**0.033**
T2 + T3	17	0.30 (0.08−1.19)	0.087	17	0.39 (0.09−1.66)	0.202
N stage						
N0	26	0.22 (0.05−1.03)	0.055	26	0.22 (0.05−1.03)	0.055
N1	5	-	-	5	-	-
N0 + N1	31	0.25 (0.07−0.90)	**0.034**	31	0.27 (0.07−0.99)	**0.049**
M stage						
M0	28	0.41 (0.12−1.34)	0.14	28	0.41 (0.12−1.34)	0.14
M1	5	-	-	5	-	-
M0 + M1	33	0.28 (0.09−0.87)	**0.028**	33	0.30 (0.10−0.95)	**0.041**
TNM stage						
1	19	0.32 (0.06−1.60)	0.167	19	0.32 (0.06−1.60)	0.167
2	9	-	**-**	9	-	-
3	1	-	-	1	-	**-**
4	7	-	-	7	-	-
1 + 2	28	0.23 (0.06−0.87)	**0.03**	28	0.26 (0.07−0.96)	**0.043**
3 + 4	8	-	-	8	-	-
Gender						
Male	16	0.44 (0.08−2.27)	0.325	16	0.500 (0.09−2.76)	0.428
Female	20	0.35 (0.09−1.38)	0.135	20	0.41 (0.10−1.64)	0.205
Race						
Asian	3	-	-	3	-	-
Black	2	-	-	2	-	-
White	31	0.30 (0.09−0.94)	**0.039**	31	0.35 (0.11−1.14)	0.082
Age						
≤65	17	0.37 (0.09−1.50)	0.164	17	0.37 (0.09−1.50)	0.164
>65	19	0.24 (0.05−1.20)	0.083	19	0.33 (0.06−1.80)	0.199
BMI			-	16	-	-
≤25	10	0.33 (0.06−1.88)	0.212	10	0.42 (0.07−2.57)	0.345
>25	25	0.39 (0.10−1.55)	0.183	25	0.45 (0.11−1.83)	0.265
Histological type						
distal	2	-	**-**	2	-	**-**
perihilar	4	-	-	4	-	-
intrahepatic	30	0.35 (0.11−1.11)	0.075	30	0.38 (0.12−1.24)	0.109
CA19-9						
abnormal	16	0.91 (0.20−4.10)	0.9	16	1.17 (0.23−5.89)	0.849
normal	14	0.10 (0.01−0.90)	**0.04**	14	0.12 (0.01−1.03)	0.054
Vascular invasion						
yes	5	-	**-**	5	-	-
no	29	0.26 (0.07−0.96)	**0.042**	29	0.29 (0.08−1.07)	0.063
Perineural invasion						
Yes	7	-	**-**	7	-	-
No	26	0.19 (0.04−0.88)	**0.034**	26	0.21 (0.04−0.98)	**0.047**

**Table 3:** The correlation of ARHGAP21 and immune-related genes.

Gene Type	Gene	Spearman	
		cor	*p*
**Chemokine genes**	CXCL16	0.34	**0.043**
	CCL28	0.403	**0.015**
	CCL13	0.359	**0.031**
**Immunoinhibitory genes**	TGFBR1	0.53	**0.001**
	PDCD1LG2	0.425	**0.01**
	KIR2DL1	−0.385	**0.02**
	KDR	0.381	**0.023**
	CD274	0.372	**0.026**
**Immunostimulatory genes**	VSIR	0.395	**0.018**
	TNFSF18	0.493	**0.002**
	PVR	0.484	**0.003**
	ENTPD1	0.45	**0.006**
	CD28	0.388	**0.02**

## Data Availability

The data used in the part of bioinformatics analysis in this manuscript are all from the TCGA (https://portal.gdc.cancer.gov/, accessed on 1 July 2021) and GEO (https://www.ncbi.nlm.nih.gov/geo/, accessed on 1 July 2021) online databases.

## Supplementary Materials

The following supporting information can be downloaded at: https://www.mdpi.com/article/10.3390/medicina59010139/s1, Figure S1: (A) The predictive nomogram consisted of histological type, perineural invasion, and ARHGAP21 expression was utilized to estimate the 1-year, 3-year and 5-year overall survival of CCA patients. (B) The predictive nomogram consisted of histological type, perineural invasion, and ARHGAP21 expression and was utilized to estimate the 1-year, 3-year, and 5-year disease-specific survival of CCA patients.
