# Supplementary figures and images for: ARHGAP21 Is Involved in the Carcinogenic Mechanism of Cholangiocarcinoma: A Study Based on Bioinformatic Analyses and Experimental Validation
Source: Medicina (Kaunas). 2023 Jan 10;59(1):139. doi: 10.3390/medicina59010139 (PMC9867224; doi:10.3390/medicina59010139)

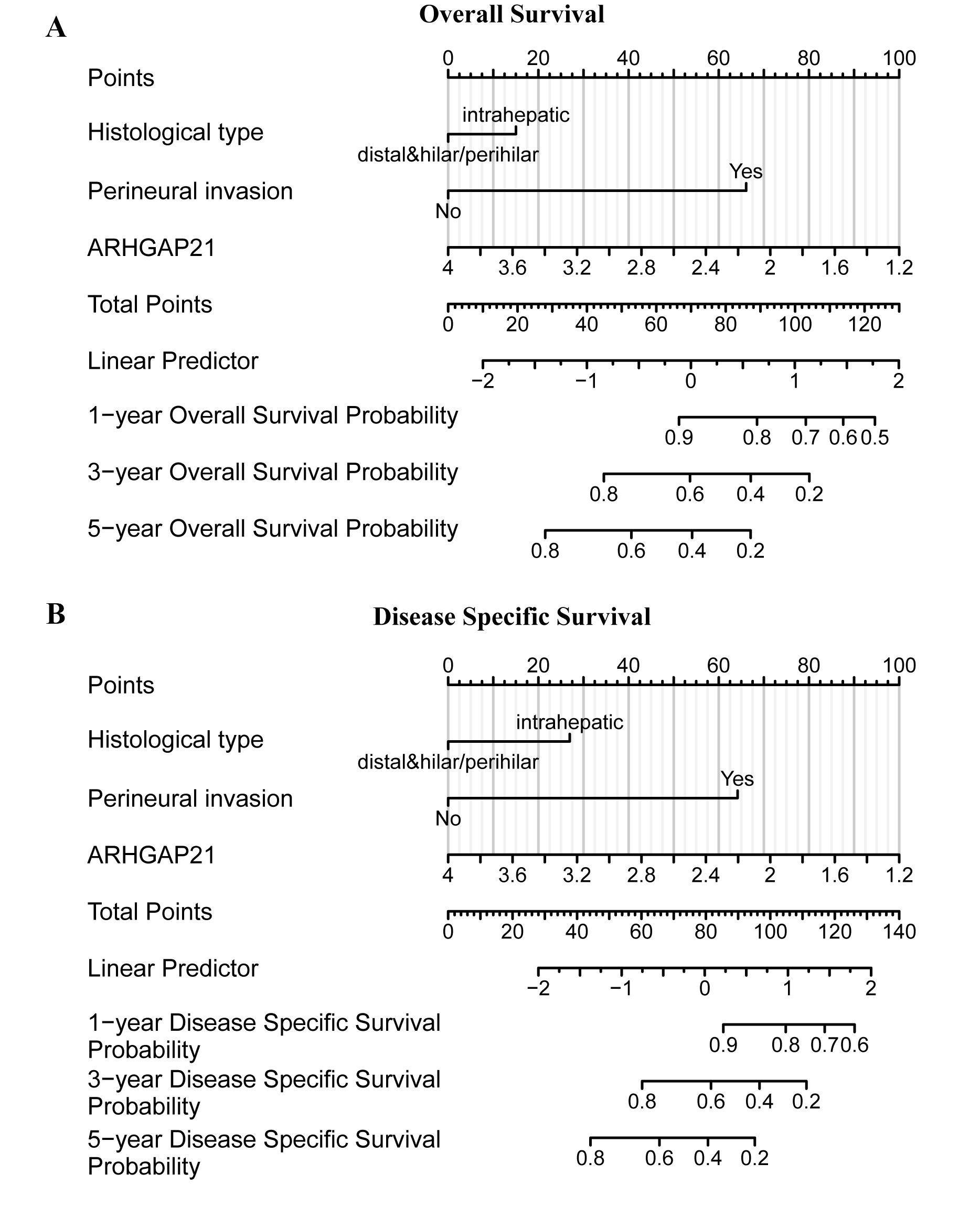

Supplement: Supplementary file 1 [file medicina-59-00139-s001.zip › medicina-2085719-supplementary.tif]
